# Supplementary material for: Increased replication of dissimilatory nitrate-reducing bacteria leads to decreased anammox bioreactor performance
Source: Microbiome. 2020 Jan 24;8:7. doi: 10.1186/s40168-020-0786-3 (PMC6982389; doi:10.1186/s40168-020-0786-3)
Supplement: Supplementary file 1 — Additional file 1: Table S1. Genome parameters for the representative bacteria from the anammox bioreactor. Temporal strain shifts in the bioreactor. Analysis of shifts between bacterial strains during the first bioreactor crash and subsequent biomass amendment. Table S2. Genome parameters for the bacterial strains related to representative bacteria from the bioreactor. Table S3 assignment of genomes to different clusters. Figure S1. Analysis of community metabolism by gene presence. Figure S2. Reduced metabolic map of the Brocadia sp. Bacterium. Electron transfer pathways in the community. Analysis of different electron donors and acceptors utilized by the anammox community members. Central carbon metabolism in the community. Analysis of carbon utilization in the anammox microbial community. Table S4. Diversity indices of the anammox community. Table S5. Synthesis and transport of metabolites in the metabolic groups. Table S6. Confidence intervals of the Log-Ratio changes for each Reference Frame genome. Table S7. Closest relative based on distance in ML tree for anammox bacterial community. Figure S3. Maximum likelihood tree of the PVC clade (Planctomycetes, Verrucomicrobia, Chlamydiae, and Lentisphaerae). Figure S4. Schematic of the anaerobic membrane bioreactor. Table S8. Composition of the synthetic wastewater feed. Supplemental methods. Refinement of the Candidatus Brocadia sp. genome. Table S9. Replication rates of genomes for each time point, calculated using the iRep program. [file 40168_2020_786_MOESM1_ESM.pdf]

Supporting information

Increased replication of dissimilatory nitrogen-reducing bacteria leads to decreased anammox bioreactor performance

Authors: Ray Keren[1]*, Jennifer E. Lawrence[2]*, Weiqin Zhuang[3], David Jenkins[1], Jillian F. Banfield[4], Lisa Alvarez-Cohen[1,5], Lijie Zhou[6]+, Ke Yu[7]+
*These authors contributed equally to the work
+Corresponding authors

Affiliations
1- Department of Civil and Environmental Engineering, University of California Berkeley, Berkeley, CA, USA
2- Tighe & Bond, Westwood, MA, USA
3- Department of Civil and Environmental Engineering, The University of Auckland, Auckland, New Zealand
4- Department of Earth and Planetary Sciences, University of California Berkeley, Berkeley, CA, USA
5- Earth and Environmental Sciences Division, Lawrence Berkeley National Laboratory, Berkeley, CA, USA
6- College of Chemistry and Environmental Engineering, Shenzhen University, Shenzhen, China
7- Shenzhen Graduate School, Peking University, Shenzhen, China

Table S1 Genome parameters for the representative bacteria from the anammox bioreactor

Genome	Genome code (ggkbase)	Phyla	Genome length (bp)	GC%	Contigs	ORFs	Completeness (%)
LAC_ACD01	anamox1_Acidimicrobiia_62_4_curated	*Acidobacteria*	298722	58.08	173	496	65.54
LAC_ACD02	anamox1_Acidobacteria_62_5_curated	*Acidobacteria*	3111074	61.59	565	3251	65.54
LAC_ACD03	anamox1_Pyrinomonas_methylaliphatogenes_54_8_curated	*Acidobacteria*	2071852	54.23	87	1989	95.69
LAC_ACD04	anamox3_Acidobacteria_71_4_curated	*Acidobacteria*	1334157	69.62	772	1759	39.71
LAC_ACD05	LAC_NA06_Candidatus_Solibacter_usitatus_62_12_curated	*Acidobacteria*	4513182	61.85	167	4021	91.38
LAC_ACD06	LAC_NA07_Acidobacteria_70_38_curated	*Acidobacteria*	3262818	69.91	40	2917	94.83
LAC_ACD07	LAC_NA07_Candidatus_Solibacter_usitatus_59_6_curated	*Acidobacteria*	1779371	57.94	742	2242	56.02
LAC_ACT01	anamox2_Actinobacteria_65_5_curated	*Actinobacteria*	2799571	63.61	662	3147	67.46
LAC_ACT02	LAC_NA07_Actinobacteria_71_14_curated	*Actinobacteria*	3047960	70.65	64	3117	93.97
LAC_ACT03	LAC_NA07_Actinobacteria_74_18_curated	*Actinobacteria*	1235923	73.53	141	1261	64.86
LAC_ACT04	LAC_NA07_Actinotalea_fermentans_75_19_curated	*Actinobacteria*	2905150	75.39	31	2745	100
LAC_ARCH01	anamox2_Methanosarcina_thermophila_41_9_curated	*Euryarchaeota*	2959285	41.19	82	2703	35.55
LAC_ARM01	LAC_NA06_Fimbriimonas_ginsengisoli_61_14_curated	*Armatimonadetes*	2673573	61.04	54	2542	88.45
LAC_BAC01	anamox1_Bacteria_33_9_curated	*Microgenomates*	775494	32.38	45	793	70.85
LAC_BAC02	anamox1_Bacteria_45_8_curated	*Ignavibacteria*	2579669	45	166	2221	87.77
LAC_BAC03	anamox1_Bacteria_50_18_curated	*TM7*	820566	49.55	22	874	77.9
LAC_BAC04	anamox1_Bacteria_53_17_curated	*Deltaproteobacteria*	3465172	53.24	12	2895	100
LAC_BAC05	anamox1_Bacteria_55_18_curated	Bacteria	4100779	54.56	216	3365	98.28
LAC_BAC06	anamox1_Bacteria_57_32_curated	*Lentisphaerae*	2939326	56.91	76	2571	95.69
LAC_BAC07	anamox1_Bacteria_57_5_curated	*Chloroflexi*	3332363	56.3	813	3846	68.62
LAC_BAC08	anamox1_Bacteria_57_9_curated	*Planctomycetes*	3469651	57.28	74	3012	93.65
LAC_BAC09	anamox1_Bacteria_64_7_curated	*Planctomycetes*	3988790	63.42	306	3679	93.03
LAC_BAC10	anamox1_Bacteria_65_5_curated	*Planctomycetes*	2089721	64.54	434	2237	51.28
LAC_BAC11	anamox1_Bacteria_72_15_curated	*Acidobacteria*	2937143	72.45	24	2507	87.93
LAC_BAC12	anamox2_Bacteria_34_5_curated	*Microgenomates*	434538	33.34	88	508	50.78
LAC_BAC13	anamox2_Bacteria_61_6_curated	*Armatimonadetes*	2415824	60.87	385	2646	65.91
LAC_BAC14	anamox2_Bacteria_68_6_curated	Bacteria	2291336	67.96	460	2621	64.03
LAC_BAC15	anamox3_Bacteria_50_5_curated	*Ignavibacteria*	1042006	49.54	203	1076	42.87
LAC_BAC16	anamox3_Bacteria_66_7_curated	*Verrucomicrobia*	2266342	65.35	286	2150	81.9
LAC_BAC17	anamox3_Bacteria_67_13_curated	Bacteria	1642722	66.5	47	1442	67.24
LAC_BAC18	anamox3_Bacteria_67_15_curated	*Chloroflexi*	2765727	66.48	144	2980	95.69
LAC_BAC19	anamox4_Bacteria_63_7_curated	*Chloroflexi*	3451341	62.64	351	3276	93.1
LAC_BAC20	anamox4_Bacteria_69_43_curated	*Deltaproteobacteria*	3827900	69.37	15	3388	86.21
LAC_BAC21	LAC_NA06_Bacteria_60_18_curated	*Deltaproteobacteria*	3689918	59.98	50	3221	94.83
LAC_BAC22	LAC_NA07_Bacteria_38_171_curated	*Ignavibacteria*	2383463	37.56	22	2157	98.28
LAC_BAC23	LAC_NA07_Bacteria_70_305_curated	*Planctomycetes*	2792777	70.13	54	2465	98.28

LAC_BAC24	LAC_NA07_Bacteria_71_12_curated	*Chloroflexi*	2770642	70.73	206	2877	81.41
LAC_BACT01	anamox1_Bacteroidetes_38_5_curated	*Bacteroidetes*	1414251	37.34	293	1501	62.96
LAC_BACT02	anamox1_Bacteroidetes_39_16_curated	*Bacteroidetes*	2896628	38.84	40	2472	100
LAC_BACT03	anamox1_Bacteroidetes_63_11_curated	*Bacteroidetes*	3356307	63.21	73	2915	100
LAC_BACT04	anamox1_Sphingobacteriales_42_27_curated	*Bacteroidetes*	3490519	42	95	3068	100
LAC_BACT05	anamox1_Sphingobacteriales_43_8_curated	*Bacteroidetes*	3156182	42.95	130	2826	97.41
LAC_BACT06	anamox2_BJP_IG2103_Bacteroidetes_37_22_46_7_curated	*Bacteroidetes*	2490972	45.52	190	2226	93.1
LAC_BACT07	anamox2_Sphingobacteriales_41_11_curated	*Bacteroidetes*	2768577	41.16	129	2490	87.07
LAC_BACT08	anamox3_Bacteroidetes_39_15_curated	*Bacteroidetes*	2533542	39.26	23	2188	100
LAC_BACT09	anamox3_Burkholderiales_71_6_curated	*Bacteroidetes*	1367674	69.86	648	1904	32.85
LAC_BACT10	anamox3_Sphingobacteriales_44_6_curated	*Bacteroidetes*	3033899	42.77	512	2768	73.9
LAC_BACT11	anamox3_Sphingobacteriales_50_9_curated	*Bacteroidetes*	4305233	49.73	216	2999	99.14
LAC_BACT12	anamox4_Bacteroidetes_40_74_curated	*Bacteroidetes*	2627547	39.91	15	2219	100
LAC_BACT13	LAC_NA06_Bacteroidetes_30_9_curated	*Bacteroidetes*	2233050	29.92	225	2055	85.06
LAC_CHLX01	anamox1_Bacteria_56_37_curated	*Chloroflexi*	4970250	55.88	43	4404	94.83
LAC_CHLX02	anamox1_Chloroflexi_52_59_curated	*Chloroflexi*	2864537	52.47	96	2759	100
LAC_CHLX03	anamox2_Chloroflexi_60_8_curated	*Chloroflexi*	2023353	60.06	111	1885	47.49
LAC_CHLX04	anamox3_Chloroflexi_59_6_curated	*Chloroflexi*	1407767	53.68	351	1507	40.22
LAC_CHLX05	anamox3_Chloroflexi_68_6_curated	*Chloroflexi*	2294191	67.35	479	2330	53.92
LAC_CHLX06	anamox4_Chloroflexi_66_15_curated	*Chloroflexi*	4383590	65.96	133	3602	91.22
LAC_CHLX07	LAC_NA06_Anaerolineales_42_27_curated	*Chloroflexi*	2409642	41.78	210	2380	93.1
LAC_CHLX08	LAC_NA06_Chloroflexi_57_14_curated	*Chloroflexi*	2719486	57.25	159	2567	86.91
LAC_CHLX09	LAC_NA06_sub_Chloroflexi_59_14_curated	*Chloroflexi*	2594752	59.45	164	2359	91.22
LAC_CHLX10	LAC_NA06_sub_Chloroflexi_61_22_curated	*Chloroflexi*	2755473	61.22	170	2707	96.55
LAC_CHLX11	LAC_NA07_Caldilinea_aerophila_61_12_curated	*Chloroflexi*	3885866	60.59	83	3168	80.88
LAC_CHLX12	LAC_NA07_Chloroflexi_57_23_curated	*Chloroflexi*	6358497	57.22	93	5201	89.66
LAC_CHLX13	LAC_NA07_Chloroflexi_57_9_curated	*Chloroflexi*	2081669	55.57	325	2239	71.76
LAC_CHLX14	LAC_NA07_Chloroflexi_58_12_curated	*Chloroflexi*	2711149	57.43	154	2459	60.42
LAC_CHLX15	LAC_NA07_Chloroflexi_60_59_curated	*Chloroflexi*	3858643	60.1	13	3478	94.83
LAC_CHLX16	LAC_NA07_Chloroflexi_65_58_curated	*Chloroflexi*	3316034	65.08	327	3017	87.93
LAC_CHLX17	LAC_NA07_Chloroflexi_67_63_curated	*Chloroflexi*	3446202	66.61	65	2852	96.55
LAC_CHLX18	LAC_NA07_RBG_16_RIF_CHLX_72_14_curated_75_20_curated	*Chloroflexi*	2506614	74.69	63	2330	91.38
LAC_CLO01	anamox1_Candidatus_Cloacimonas_acidaminovorans_38_6_curated	Bacteria	1192038	35.28	246	1132	56.47
LAC_D-T01	anamox1_Truepera_radiovictrix_71_5_curated	*Deinococcus Thermus*	435656	69.12	317	694	24.49
LAC_D-T02	LAC_NA07_Truepera_radiovictrix_72_29_curated	*Deinococcus Thermus*	1353035	72.21	165	1373	82.6
LAC_DADA01	LAC_NA06_RIFCSPHIGHO2_12_FULL_Dadabacteria_53_21_curated_58_6_curated	*Dadabacteria*	869333	55.01	539	1358	33.68
LAC_GMT01	anamox1_Gemmatimonas_aurantiaca_57_6_curated	*Gemmatimonadetes*	2631895	56.94	255	2549	91.22
LAC_IGN01	anamox1_RBG_16_Ignavibacteria_36_9_curated_35_5_curated	*Ignavibacteria*	1163158	34.03	347	1374	70.14
LAC_IGN02	anamox2_Ignavibacteriales_33_72_curated	*Ignavibacteria*	3331053	33.19	161	2995	98.28

LAC_IGN03	anamox2_Ignavibacteriales_33_9_curated	*Ignavibacteria*	2936140	33.09	166	2753	86.05
LAC_IGN04	anamox2_Ignavibacteriales_41_12_curated	*Ignavibacteria*	3156563	41.32	42	2805	96.55
LAC_IGN05	anamox2_sub_Ignavibacterium_album_33_16_curated	*Ignavibacteria*	3391206	33.43	195	3029	97.81
LAC_IGN06	anamox3_BJP_IG2069_Ignavibacteriae_38_11_30_7_curated	*Ignavibacteria*	1803433	30.38	132	1600	73.51
LAC_IGN07	anamox3_sub_Ignavibacteriales_42_14_curated	*Ignavibacteria*	3163923	42.21	22	2498	96.55
LAC_MIC01	anamox2_Microgenomates_45_6_curated	*Microgenomates*	740215	44.28	99	904	73.35
LAC_MIC02	anamox2_Microgenomates_49_6_curated	*Microgenomates*	950204	49.23	109	1082	68.42
LAC_MIC03	anamox2_Roizmanbacteria_38_11_curated	*Microgenomates*	663231	37.62	14	693	58.31
LAC_MIC04	anamox4_Microgenomates_48_8_curated	*Microgenomates*	999881	47.64	49	1055	80.02
LAC_MIC05	LAC_NA06_Microgenomates_41_17_curated	*Microgenomates*	1110978	41.17	7	1216	80.88
LAC_MIC06	LAC_NA07_Roizmannbacteria_52_60_curated	*Microgenomates*	957307	52.33	6	1013	73.98
LAC_NIT01	anamox4_Candidatus_Nitrospira_defluvii_60_9_curated	*Microgenomates*	3085099	60.3	94	3084	95.69
LAC_OMN01	anamox3_Omnitrophica_63_14_curated	*Omnitrophica*	365510	62.81	4	402	25.86
LAC_PLT01	anamox1_Planctomycetia_64_8_curated	*Planctomycetes*	4138393	63.58	783	3420	87.62
LAC_PLT02	anamox4_sub_Candidatus_Brocadia_sinica_42_75_curated	*Planctomycetes*	3107335	42.29	64	2859	98.28
LAC_PROT01	anamox1_Betaproteobacteria_69_7_curated	*Betaproteobacteria*	2106569	66.39	531	2636	66.55
LAC_PROT02	anamox1_Burkholderiales_70_40_curated	*Betaproteobacteria*	3601855	70.09	60	3356	100
LAC_PROT03	anamox1_Burkholderiales_71_17_curated	*Betaproteobacteria*	3639459	70.81	114	3448	99.22
LAC_PROT04	anamox1_Gammaproteobacteria_64_6_curated	*Gammaproteobacteria*	2447777	64.02	391	2594	84.25
LAC_PROT05	anamox1_Lysobacter_67_10_curated	*Gammaproteobacteria*	783854	66.76	125	840	34.64
LAC_PROT06	anamox1_Nitrosomonas_europaea_50_14_curated	*Betaproteobacteria*	2128781	50.43	37	2006	98.28
LAC_PROT07	anamox1_Proteobacteria_65_15_curated	*Gammaproteobacteria*	2240508	65.45	348	2476	58.59
LAC_PROT08	anamox1_Proteobacteria_67_8_curated	*Gammaproteobacteria*	2912885	66.93	203	2910	81.27
LAC_PROT09	anamox1_Rhodocyclales_69_13_curated	*Betaproteobacteria*	2263347	68.53	185	2364	96.55
LAC_PROT10	anamox2_Burkholderiales_67_5_curated	*Betaproteobacteria*	1246117	66.28	298	1434	38.05
LAC_PROT11	anamox2_Burkholderiales_68_9_curated	*Betaproteobacteria*	3527974	68.25	181	3452	91.22
LAC_PROT12	anamox2_Burkholderiales_70_13_curated	*Betaproteobacteria*	3074974	70.33	39	2841	84.48
LAC_PROT13	anamox2_Gammaproteobacteria_67_23_curated	*Gammaproteobacteria*	2663638	67.06	116	2584	79.31
LAC_PROT14	anamox2_Hydrogenophilales_66_19_curated	*Betaproteobacteria*	2257110	65.94	122	2331	91.95
LAC_PROT15	anamox2_Myxococcales_71_5_curated	*Deltaproteobacteria*	1434218	70.21	495	1687	30.21
LAC_PROT16	anamox2_Rhizobiales_67_45_curated	*Alphaproteobacteria*	4837226	66.56	24	4668	100
LAC_PROT17	anamox2_Xanthomonadales_68_9_curated	*Gammaproteobacteria*	1242229	67.63	21	1168	50
LAC_PROT18	anamox2_Xanthomonadales_70_8_curated	*Gammaproteobacteria*	2605543	68.54	346	2555	82.76
LAC_PROT19	anamox3_Nitrosomonas_europaea_51_28_curated	*Betaproteobacteria*	2372894	50.54	93	2302	98.12
LAC_PROT20	anamox3_Proteobacteria_68_11_curated	*Gammaproteobacteria*	3218375	68.07	66	3078	100
LAC_PROT21	anamox4_Alphaproteobacteria_58_14_curated	*Alphaproteobacteria*	2593178	57.86	5	2558	98.28
LAC_PROT22	anamox4_Gammaproteobacteria_67_14_curated	*Gammaproteobacteria*	3265689	66.91	73	3049	98.28
LAC_PROT23	anamox4_Nitrosomonas_eutropha_48_11_curated	*Betaproteobacteria*	1880399	48.46	103	1862	94.83
LAC_PROT24	LAC_NA06_Betaproteobacteria_71_7_curated	*Betaproteobacteria*	4060980	69.45	1841	5695	69.75

LAC_PROT25	LAC_NA06_Burkholderiales_73_13_curated	*Betaproteobacteria*	3708868	72.67	335	3469	94.51
LAC_PROT26	LAC_NA06_Rhizobiales_66_24_curated	*Alphaproteobacteria*	3228463	66.32	148	3270	98.28
LAC_PROT27	LAC_NA07_Burkholderiales_70_312_curated	*Betaproteobacteria*	2688511	69.75	290	2827	93.32
LAC_PROT28	LAC_NA07_Proteobacteria_68_32_curated	*Gammaproteobacteria*	2747459	68.4	14	2541	98.28
LAC_PROT29	LAC_NA07_Rhodobacterales_68_7_curated	*Alphaproteobacteria*	1308110	67.47	473	1651	52.93
LAC_PROT30	LAC_NA07_Rhodocyclales_67_14_curated	*Betaproteobacteria*	2723825	66.65	212	2909	92.95
LAC_SCH01	LAC_NA06_Candidatus_Saccharibacteria_40_6_curated	*TM7*	463567	39.93	148	580	58.46
LAC_SPR01	anamox1_Turneriella_parva_44_7_curated	*Spirochaetes*	2667359	44.12	240	2748	92.24
LAC_VER01	anamox2_Verrucomicrobia_58_8_curated	*Verrucomicrobia*	3592680	57.68	250	3293	87.93
LAC_VER02	anamox2_Verrucomicrobia_62_8_curated	*Verrucomicrobia*	3685167	61.78	161	3134	98.28
LAC_VER03	anamox3_Opitutus_terrae_67_4_curated	*Verrucomicrobia*	780321	65.72	503	1046	24.42
LAC_VER04	anamox3_Pedosphaera_parvula_66_5_curated	*Verrucomicrobia*	1233438	65.98	543	1384	56
LAC_VER05	anamox3_Verrucomicrobia_59_12_curated	*Verrucomicrobia*	2712092	59.27	50	2592	96.55

Temporal strain shifts in the bioreactor. Between Days 82 and 328, we binned 2-3 partial bins of the *Brocadia* sp (at each time point). These partial bins were separated by their coverage peaks, indicating strain variation. The presence of multiple strains at some of the time points was verified by the dereplication process. The *Brocadia* sp. cluster contained complete genome bins of the two strains: anamox1_Candidatus_Brocadia_sinica_42_73_curated (strain 1) and LAC_PLT02 (strain 2 and the representative genome of the anammox bacterium). The first genome was binned from the source community and can be found up to Day 166 in the reactor. The second genome is represented from Day 284 and onwards. This strain is the only *Brocadia* sp. bacterium (and anammox bacterium) present at stable state in Day 437.

Apart from *Brocadia* sp., we observed strain shift in eight other bacteria around the same time. Several are noteworthy: LAC_IGN05, which shifted with anamox4_Ignavibacterium_album_34_177_curated, was the most dominant bacteria during the destabilization period. Apart from this bacterium, three other bacteria were also associated with the destabilization. It seems that the biomass amendment introduced not only an anammox bacterium that came to dominate the community, but also strong destabilizing bacteria.

anamox2_sub_Burkholderiales_70_25_curated shifted into LAC_PROT27, and LAC_MIC02 shifted to LAC_NA07_Microgenomates_50_23_curated. The first bacterium was one of three bacteria consistently in the top ten abundant bacteria. Its abundance was highest when the anammox bioreactor was performing well. The second bacterium exhibited a three-fold increase in abundance between Days 82 and 437. These two bacteria were left outliers (on the x axis) of Group D in the nMDS (Figure 4B). While the pairwise correlation between the two bacteria is not the highest among Group D, the results of the nMDS and strain shift might suggest that the two are a host-CPR pair.

Table S2 Genome parameters for the strain bacteria from the anammox reactor

**Genome**	**Taxonomy (ggKbase)**	**length (bp)**	**GC%**	**contigs**	**features**	**Completeness**
anamox1_Candidatus_Brocadia_sinica_42_73_curated	*Candidatus Brocadia sinica, Candidatus Brocadia, Candidatus Brocadiales, Planctomycetia, Planctomycetes, Bacteria*	3182191	42.3	140	2861	98.28
anamox2_Chloroflexi_65_7_curated	*Chloroflexi, Bacteria*	2846499	64	427	2818	68.5
anamox2_sub_Burkholderiales_70_25 _curated	*Burkholderiales, Betaproteobacteria, Proteobacteria, Bacteria*	1637210	69.92	260	1745	86.21
anamox3_Actinobacteria_74_6_curated	*Actinobacteria, Actinobacteria, Bacteria*	1306370	70.77	293	1474	33.9
anamox3_Truepera_radiovictrix_72_6 _curated	*Truepera radiovictrix, Truepera, Deinococcales, Deinococci, Deinococcus-Thermus, Bacteria*	1087782	69.47	428	1343	47.82
anamox4_Betaproteobacteria_69_7 _curated	*Betaproteobacteria, Proteobacteria, Bacteria*	2004013	69.34	569	2383	59.72
anamox4_Ignavibacterium_album_34_177_curated	*Ignavibacterium album, Ignavibacterium, Ignavibacteriales, Ignavibacteria, Ignavibacteriae, Bacteria*	1414754	33.55	464	1618	63.32
LAC_NA06_Rubrivivax_70_13_curated	*Rubrivivax, Burkholderiales, Betaproteobacteria, Proteobacteria, Bacteria*	1206889	70.2	103	1213	70.61
LAC_NA07_Microgenomates_50_23 _curated	*Microgenomates, Bacteria*	947146	49.7	96	1047	66.74

Table S3 assignment of genomes to different clusters

Genome	Abundance Group	Metabolic Group	Association grouping
LAC_ACD01	A	NA	NA
LAC_ACD02	A	NA	NA
LAC_ACD03	C	ɣ	AA
LAC_ACD04	A	NA	NA
LAC_ACD05	B	ɣ	AA
LAC_ACD06	D	ɣ	AA
LAC_ACD07	D	NA	NA
LAC_ACT01	C	NA	NA
LAC_ACT02	D	α	AA
LAC_ACT03	B	NA	NA
LAC_ACT04	D	α	AA
LAC_ARCH01	C	NA	NA
LAC_ARM01	D	ɣ	AA
LAC_BAC01	A	β	SA
LAC_BAC02	A	δ	SA
LAC_BAC03	A	β	SA
LAC_BAC04	A	ɣ	SA
LAC_BAC05	A	ɣ	SA
LAC_BAC06	A	ɣ	SA
LAC_BAC07	A	NA	NA
LAC_BAC08	A	ɣ	SA
LAC_BAC09	A	ɣ	SA
LAC_BAC10	A	NA	NA
LAC_BAC11	A	ɣ	SA
LAC_BAC12	C	NA	NA
LAC_BAC13	C	NA	NA
LAC_BAC14	C	NA	NA
LAC_BAC15	A	NA	NA
LAC_BAC16	A	ɣ	SA
LAC_BAC17	A	NA	NA
LAC_BAC18	A	α	SA
LAC_BAC19	B	α	AA
LAC_BAC20	B	ɣ	AA
LAC_BAC21	A	ɣ	AA
LAC_BAC22	D	δ	AA
LAC_BAC23	D	ɣ	AA

LAC_BAC24	B	α	AA
LAC_BACT01	A	NA	NA
LAC_BACT02	A	δ	SA
LAC_BACT03	C	δ	SA
LAC_BACT04	B	ɣ	AA
LAC_BACT05	A	ɣ	SA
LAC_BACT06	C	δ	SA
LAC_BACT07	C	ɣ	SA
LAC_BACT08	A	δ	SA
LAC_BACT09	A	NA	NA
LAC_BACT10	A	ɣ	SA
LAC_BACT11	A	δ	SA
LAC_BACT12	A	δ	AA
LAC_BACT13	B	δ	SA
LAC_CHLX01	B	α	AA
LAC_CHLX02	A	α	SA
LAC_CHLX03	C	NA	NA
LAC_CHLX04	B	NA	NA
LAC_CHLX05	A	NA	NA
LAC_CHLX06	B	α	AA
LAC_CHLX07	B	α	AA
LAC_CHLX08	D	α	AA
LAC_CHLX09	B	α	AA
LAC_CHLX10	D	α	AA
LAC_CHLX11	D	α	AA
LAC_CHLX12	D	α	AA
LAC_CHLX13	D	α	AA
LAC_CHLX14	D	NA	NA
LAC_CHLX15	D	α	AA
LAC_CHLX16	D	α	AA
LAC_CHLX17	D	α	AA
LAC_CHLX18	D	α	AA
LAC_CLO01	A	NA	NA
LAC_D-T01	B	NA	NA
LAC_D-T02	B	α	AA
LAC_DADA01	B	NA	NA
LAC_GMT01	A	ɣ	SA
LAC_IGN01	A	δ	SA
LAC_IGN02	C	δ	AA

LAC_IGN03	C	δ	SA
LAC_IGN04	C	ɣ	SA
LAC_IGN05	B	δ	AA
LAC_IGN06	B	δ	SA
LAC_IGN07	A	ɣ	AA
LAC_MIC01	D	β	AA
LAC_MIC02	D	NA	NA
LAC_MIC03	C	NA	NA
LAC_MIC04	B	β	AA
LAC_MIC05	B	β	AA
LAC_MIC06	D	β	AA
LAC_NIT01	A	ɣ	SA
LAC_OMN01	A	NA	NA
LAC_PLT01	A	ɣ	SA
LAC_PLT02	D	ɣ	AA
LAC_PROT01	B	NA	NA
LAC_PROT02	A	ε	AA
LAC_PROT03	A	ε	AA
LAC_PROT04	A	ɣ	SA
LAC_PROT05	A	NA	NA
LAC_PROT06	A	ε	SA
LAC_PROT07	A	NA	NA
LAC_PROT08	A	ε	SA
LAC_PROT09	A	ε	SA
LAC_PROT10	C	NA	NA
LAC_PROT11	C	ε	SA
LAC_PROT12	C	ε	SA
LAC_PROT13	C	ε	SA
LAC_PROT14	C	ε	SA
LAC_PROT15	C	NA	NA
LAC_PROT16	C	ε	SA
LAC_PROT17	C	NA	NA
LAC_PROT18	B	ε	AA
LAC_PROT19	A	ε	SA
LAC_PROT20	A	ε	SA
LAC_PROT21	D	ε	AA
LAC_PROT22	B	ε	AA
LAC_PROT23	A	ε	AA
LAC_PROT24	B	NA	NA

LAC_PROT25	B	ε	SA
LAC_PROT26	D	ε	AA
LAC_PROT27	D	ε	AA
LAC_PROT28	D	ε	AA
LAC_PROT29	D	NA	NA
LAC_PROT30	C	ε	AA
LAC_SCH01	B	NA	NA
LAC_SPR01	A	ɣ	SA
LAC_VER01	C	ɣ	SA
LAC_VER02	A	ɣ	SA
LAC_VER03	A	NA	NA
LAC_VER04	A	NA	NA
LAC_VER05	A	ɣ	SA

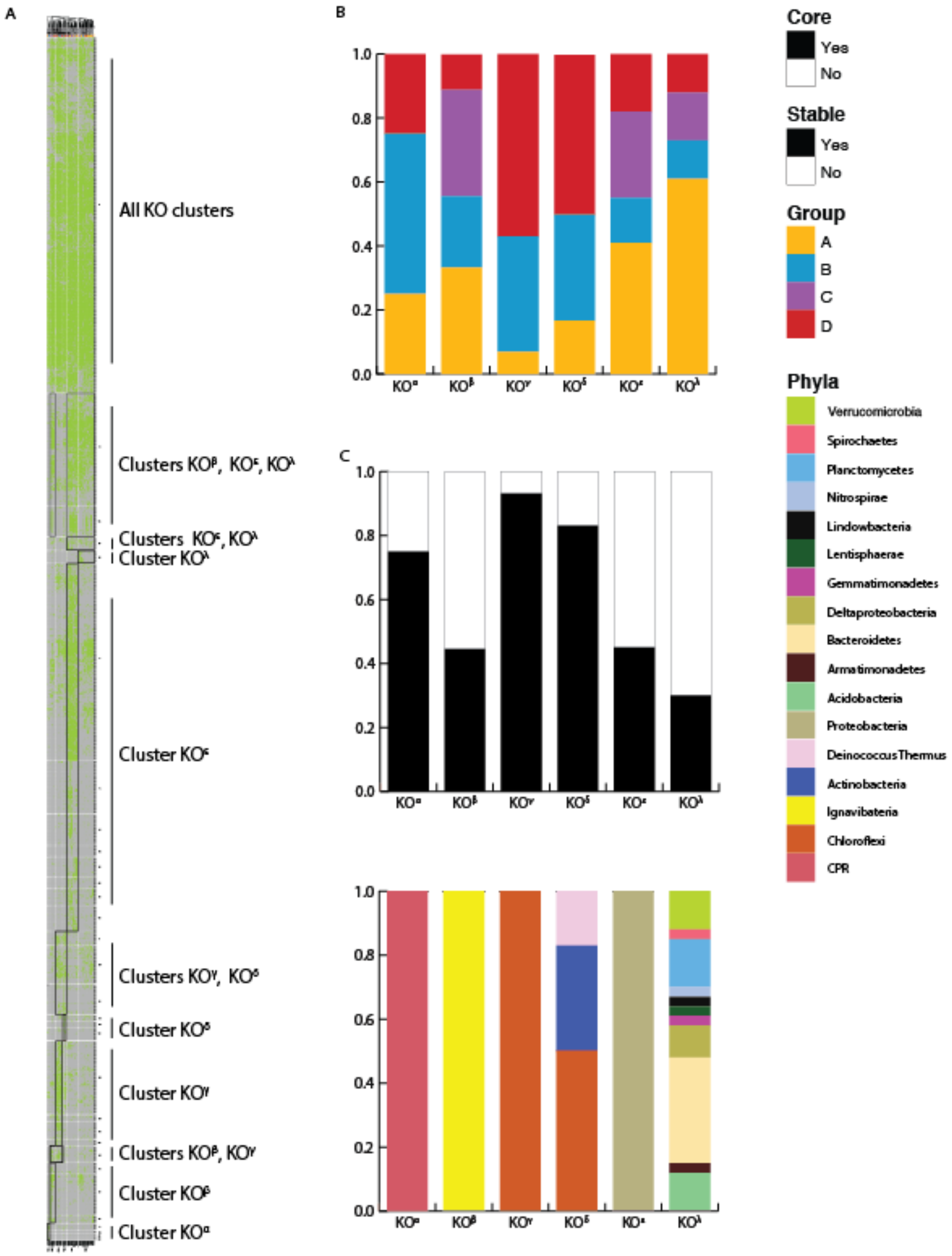

Figure S1 Analysis of community metabolism by gene presence. Genes were annotated against the KEGG database. A) A presence/absence-based clustering of both genomes and KOs using Jaccard distance and complete clustering method. The dual clustering forms "blocks" across rows and columns, and blocks that are representative of a genome cluster are presented. Black squares mark the representative blocks and on the right, the relevant cluster is mentioned. B) Fractionation of the KO cluster by abundance grouping (A-D). C) Fractionation of KO clusters by SA and AA groups. Both panels C and D indicate that clusters KOɣ and KOδ are highly associated with stable state, while cluster KOλ is the most associated with the sludge. D) Fractionation of KO clusters by taxonomy. Most KO clusters have a strong phylogenetic signal, with four of six having members of a single phylum.

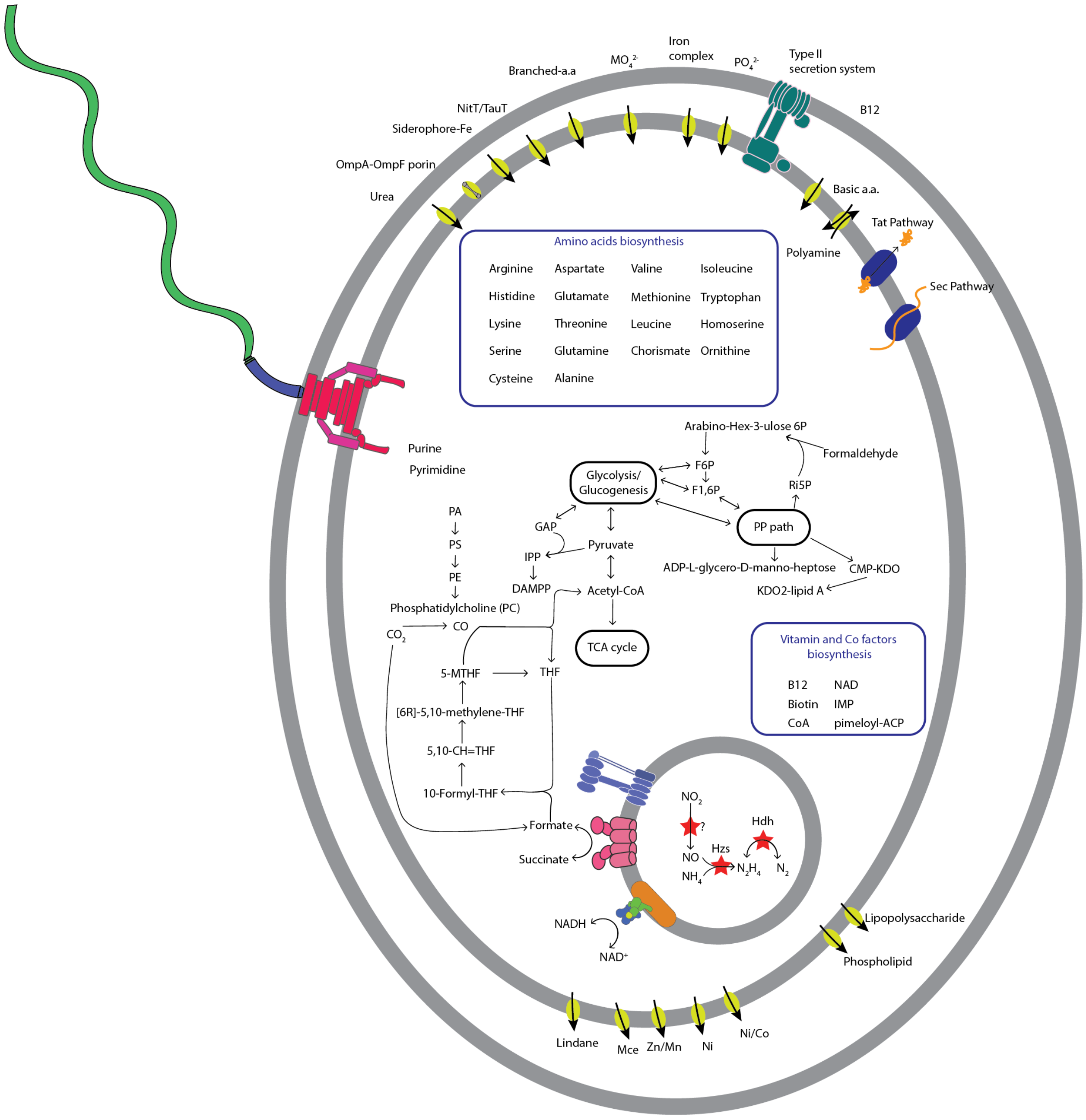

Figure S2 Reduced metabolic map of the *Brocadia* sp. Bacterium. The bacterium has the enzymes needed for the anammox process, and fixes carbon via the Wood-Ljungdahl pathway. It can synthesize 18/20 essential aa., and is the only bacterium in the reactor that can synthesize vitamin B12.

Electron transfer pathways in the community. Apart from nitrogen reduction, another common anaerobic respiration pathway within the community is acetate fermentation (genes detected in 60% of the genomes). This process was more common in AA (69%) bacteria than in SA (51%) bacteria. Ni-Fe Hydrogenase was present in 31% of the genomes, but was most common among the *Chloroflexi* of group α (87% and 48% of all occurrences of hydrogenases found) (Figure 6B).

The majority of bacteria in the bioreactor are potentially facultative aerobes (58%). All had high affinity complex IV, which differed between AA and SA bacteria. In the AA bacteria, the bd type was found in all aerobic members of group α (one also has a cbb3 type) and the *Ignavibacteria*, and the cbb3 type occurred mostly in *Proteobacteria*. For the SA bacteria, the cbb3 type was found in 24/25 aerobes and the bd-type was only found in 6/25 (only in one bacterium it is the sole variant). Complex III, which is also essential to aerobic respiration, was only found in 14 *Proteobacteria*, one *Actinobacterium*, and one *Chloroflexi*. It is possible that other bacteria have an alternative Complex III [30] that cannot be found by current KEGG annotations. Complexes I/II were found in nearly all of the bacteria, except CPR. Only five bacteria lacked the F-type ATPase; two had the V-type ATPase instead.

Central carbon metabolism in the community. It is likely that nearly all bacteria (98%) in the bioreactor can oxidize sugar by glycolysis (Figure 6A and E). Fewer bacteria (69%) have the pentose phosphate pathway (PPP). Acetyl-CoA could be synthesized from pyruvate (90% general, 98% AA, and 81% SA), or by beta-oxidation (49% general, 57% AA, and 43% SA). The majority of bacteria have the full TCA cycle (84%, or 88% after excluding CPR). A possible major carbon source for the bacteria in the bioreactor are amino acids (aa.), with 95% being able to incorporate aa. into their central carbon metabolism. The most common aa. (aspartate) could be converted into oxaloacetate and fed into the TCA cycle. Three aa. (serine, alanine, and cysteine) could be converted to pyruvate. Of these, only cysteine conversion is unidirectional, so aa., as a carbon source, cannot be ascertained. Group α had additional genes that support a reliance on proteins for their metabolism (Figure 6B). They also had a set of peptidases, as well as multiple transporters covering all forms of aa., peptides, and polyamines.

Some metabolic groups could use aa. as precursors for synthesis of other metabolites. Glutamate and histidine could be converted to PRPP, and glutamine to pyrimidines (Figure 6A). Groups ɣ and ε could use aspartate to synthesize NAD^+, and glutamine to synthesis IMP (Figure 6C). NAD^+ and IMP could not be synthesized by all of the bacteria, indicating that there are potential metabolic interdependencies in the community. Members of group ε (Figure 6D) could use leucine as a precursor to acetyl-CoA, lysine for acetoacetyl-CoA, glutamate for glutathione, and chorismate for ubiquinone. The last two could only be synthesized by group ε, indicating additional potential metabolic interdependencies in the community.

Table S4 Diversity indices of the anammox community

A) Alpha diversity (Shannon) for pairwise timepoints

	Day 0	Day 82	Day 166	Day 284	Day 328	Day 437
**Day 0**	29.23	22.81	27.89	14.28	18.59	18.59
**Day 82**	22.81	17.81	21.77	11.15	14.52	14.52
**Day 166**	27.89	21.77	26.62	13.63	17.75	17.75
**Day 284**	14.28	11.15	13.63	6.98	9.09	9.09
**Day 328**	24.17	18.87	23.07	11.81	15.38	15.38
**Day 437**	18.59	14.52	17.75	9.09	11.83	11.83

B) Beta diversity for pairwise timepoints

	DAY 0	DAY 82	DAY 166	DAY 284	DAY 328	DAY 437
**DAY 0**	1.00	1.41	1.08	1.39	1.55	1.55
**DAY 82**	1.41	1.00	1.21	1.14	1.18	1.18
**DAY 166**	1.08	1.21	1.00	1.17	1.31	1.31
**DAY 284**	1.39	1.14	1.17	1.00	1.12	1.12
**DAY 328**	1.37	1.17	1.20	1.14	1.11	1.11
**DAY 437**	1.55	1.18	1.31	1.12	1.00	1.00

Table S5 Synthesis and transport of metabolites in the MO groups. *Brocadia sp.* is considered separately from its MO grouping. Green denotes >50% of the group have the ability to synthesis/transport. Yellow denotes >40% of the group have the ability to synthesis/transport. Red denotes <40% of the group have the ability to synthesis/transport.

A) Synthesis of aa.

Module	Amino acid	MOα	MOγ	Brocadia	MOβ	MOδ	MOε
By KO	arginine	Green	Green	Green	Red	Red	Green
M00026	histidine	Green	Green	Green	Red	Red	Green
M00016/M00526	lysine	Green	Green	Green	Red	Red	Green
By KO	aspartate	Green	Green	Green	Green	Green	Green
By KO	glutamate	Green	Green	Green	Red	Green	Green
M00020	serine	Red	Green	Green	Red	Green	Green
M00018	threonine	Green	Green	Green	Red	Red	Green
By KO	asparagine	Green	Green	Green	Red	Green	Green
By KO	glutamine	Green	Green	Green	Red	Green	Green
M00021/M00338	cysteine	Green	Yellow	Green	Red	Green	Green
By KO	glycine	Green	Green	Green	Green	Green	Green
M00015	proline	Red	Red	Red	Red	Red	Green
By KO	alanine	Green	Green	Green	Red	Green	Green
M00019	valine	Green	Yellow	Green	Red	Red	Green
M00019/M00570	isoleucine	Green	Yellow	Green	Red	Red	Green
M00432+ KO	leucine	Green	Green	Green	Red	Red	Green
M00017	methionine	Red	Yellow	Red	Red	Red	Green
M00024	phenylalanine	Red	Red	Red	Red	Red	Yellow
M00025	tyrosine	Red	Red	Red	Red	Red	Red
M00023	tryptophan	Green	Green	Green	Red	Red	Green

* 39% of MOα can synthesis serine and proline
** 30% of MOε can synthesis tyrosine
While MOα have few a.a. auxotrophies they have multiple a.a. transporters (as well as peptides). They also have an array of proteases. This indicates that they might first use external a.a. sources before spending energy on synthesizing their own.

B) Synthesis of vitamins/cofactors

Module	Vitamin/Cofactor	MOα	MOγ	Brocadia	MOβ	MOδ	MOε
M00115	NAD	Red	Green	Green	Red	Green	Green
M00116	Menaquinone	Red	Red	Red	Red	Green	Red
M00117	Ubiquinone	Red	Red	Red	Red	Red	Green
M00118	Glutathione	Red	Red	Red	Red	Red	Green
M00120	Coenzyme A	Green	Green	Green	Red	Green	Green
M00123	Biotin (from pimeloyl)	Red	Red	Red	Red	Red	Green
M00124	Pyridoxal	Red	Yellow	Green	Red	Green	Green
M00126	Tetrahydrofolate	Red	Yellow	Red	Red	Green	Green
M00140	C1-unit interconversion (bugs)	Green	Green	Green	Green	Green	Green
M00141	C1-unit interconversion, (euks)	Green	Red	Red	Red	Red	Red
M00572	Pimeloyl-ACP	Yellow	Green	Green	Red	Red	Green
M00573	Biotin (from long chain acyl-ACP)	Red	Red	Red	Red	Red	Green
M00577	Biotin (form pimelate)	Red	Red	Red	Red	Red	Green
By KO	Vitamin B12	Red	Red	Green	Red	Red	Red

* 39% of MOα can synthesize menaquinone
** 33% of MOγ can synthesize Biotin (per module) and menaquinone

B) Synthesis of lipids/fatty acids

module	metabolite/process	MOα	MOγ	Brocadia	MOβ	MOδ	MOε
M00082	F.a. initiation	Red	Red	Green	Red	Green	Green
M00083	F.a. elongation	Red	Green	Red	Red	Green	Green
M00086	acyl-CoA	Green	Green	Red	Red	Green	Green
M00087	Beta oxidation	Green	Red	Red	Red	Red	Green
M00089	Triacylglycerol	Red	Red	Red	Red	Red	Yellow
M00091	Phosphatidylcholine	Red	Red	Green	Red	Red	Yellow
M00092	Phosphatidylethanolamine (from PA)	Red	Red	Red	Red	Red	Green
M00098	Acylglycerol	Red	Red	Red	Red	Red	Red
M00060	KDO2-lipid A	Red	Green	Green	Red	Green	Green
M00063	CMP-KDO	Red	Green	Green	Red	Green	Green
M00064	ADP-L-glycero-D-manno-heptose	Red	Red	Green	Red	Green	Yellow
M00080	O-antigen	Red	Red	Red	Red	Red	Red

* nine MOα and six MOγ (all that are not complete) have 50% completeness for f.a initiation
** 33% of MOγ can initiate f.a. synthesis, synthesize phosphatidylcholine and ADP-L-glycero-D-manno-heptose, and have complete beta-oxidation
*** 30% of MOε can synthesize acylglycerol

C) Transport systems

	Associated bacteria	Sludge associated bacteria	Anammox bacterium	CPR Bacteria	Ignavibacteriae/Bacteroides	Proteobacteria
metabolite	MOα	MOɣ	Brocadia	MOβ	MOδ	MOε
Capsular polysaccharide	red	red	red	red	red	red
Lipopolysaccharide	green	green	green	green	red	red
Lipooligosaccharide	red	red	red	red	red	red
Sodium	red	red	red	red	red	red
ABC-2 type	green	green	green	green	green	green
Lipoprotein-releasing system	red	red	red	red	green	green
Cell division	red	yellow	red	green	green	green
Putative ABC	green	green	green	red	green	green
Heme	green	green	red	red	green	green
Lipopolysaccharide export system	red	green	green	red	green	green
Iron complex	green	green	green	red	red	green
Vitamin B12	red	red	red	red	red	red
Zinc	red	red	red	red	green	red
Manganese/iron	green	red	red	red	red	red
Putative zinc/manganese	red	red	green	red	red	red
Cobalt/nickel	red	red	green	red	red	red
Nickel	red	red	green	red	red	red
Manganese/zinc/iron	red	red	red	red	red	red
Biotin	red	red	red	red	red	red
Energy-coupling factor	green	red	red	red	red	red
Sulfate	red	red	red	red	red	red
Tungstate	green	red	red	red	red	yellow
NitT/TauT family	green	green	green	red	red	green
Molybdate	green	red	green	red	red	green
Iron(III)	green	red	red	red	red	green
Thiamine	green	red	red	red	red	red
Putative spermidine/putrescine	green	red	red	red	red	red
Glycine betaine/proline	red	red	red	red	red	red
Osmoprotectant	red	red	red	red	red	red
Spermidine/putrescine	green	red	red	red	red	red
Putrescine	red	red	red	red	red	yellow
Peptides/nickel	green	green	red	red	green	green
Microcin C	red	red	red	red	red	red
Oligopeptide	red	red	red	red	red	red
Phosphate	green	red	green	red	red	green
Phosphonate	green	red	red	red	red	red
Glutamate/aspartate	red	red	red	red	red	red
General L-amino acid	red	red	red	red	red	red
Putative polar amino acid	green	red	red	red	red	red
Branched-chain amino acid	green	red	red	red	red	green
Raffinose/stachyose/melibiose	green	red	red	red	red	red
Putative sn-glycerol-phosphate	red	red	red	red	red	red
alpha-Glucoside	green	red	red	red	red	red
Trehalose/maltose	red	red	red	red	red	red
Putative multiple sugar	green	green	red	red	red	red
Phospholipid	red	red	green	red	red	green
Ribose	green	red	red	red	red	red
D-Xylose	red	red	red	red	red	red
Multiple sugar	red	red	red	red	red	red
Putative simple sugar	green	red	red	red	red	red
arabinogalactan oligomer/maltooligosaccharide	green	red	red	red	red	red
Glucose/mannose	red	red	red	red	red	red
gamma-Hexachlorocyclohexane	red	green	green	red	green	green
Mce	red	green	green	red	green	green

Table S6 Confidence intervals of the Log-Ratio changes for each reference frame genome (RFg)

	Upper Limit	Lower Limit
RF1 (LAC_NA06_Anaerolineales_42_27)	-0.07005769	-0.7292107
RF2 (anamox4_Bacteria_63_7_curated)	-0.07005769	-0.7292107
RF3 (anamox4_Gammaproteobacteria_67_14_curated)	0.3940631	-0.390227
RF4 (LAC_NA07_Proteobacteria_68_32)	-0.2433609	-0.8117102

Table S7 Closest relatives based on distance in ML tree, for the anammox bacterial community

**Genome**	**Closest relative**	**Distance**	**Similarity**
**LAC_ACD01**	**Actinobacteria_bacterium_RBG_16_70_17**	**0.58**	**88.56**
**LAC_ACD02**	**LAC_ACD07**	**0.42**	**91.74**
**LAC_BAC01**	**OLB21**	**0.78**	**84.69**
**LAC_BAC02**	**LAC_BAC15**	**0.59**	**88.39**
**LAC_BAC03**	**CG10_big_fil_rev_8_21_14_0_10_Saccharibacteria_47_8**	**1.17**	**76.89**
**LAC_BAC04**	**Deltaproteobacteria_bacterium_GWA2_45_12**	**0.88**	**82.55**
**LAC_BAC05**	**OLB16**	**0.00**	**99.92**
**LAC_CHLX01**	**UTCFX4**	**0.12**	**97.66**
**LAC_BAC06**	**Bacteria_Lentisphaerae_GWF2_Lentisphaerae_57_35**	**0.66**	**87.00**
**LAC_BAC07**	**LAC_CHLX01**	**0.25**	**95.01**
**LAC_BAC08**	**LAC_BAC09**	**0.31**	**93.82**
**LAC_BAC09**	**LAC_BAC08**	**0.31**	**93.82**
**LAC_BAC10**	**Bacteria_Plantomycetes_uncultured_DG_23**	**1.36**	**73.17**
**LAC_BAC11**	**Bacteria_Acidobacteria_RBG_16_Acidobacteria_64_8**	**0.34**	**93.22**
**LAC_BACT01**	**Bacteria_BacteroidetesChlorobi_group_Bacteroidetes_Bacteroidetes_Order_III__Incertae_sedis_Thermonema_rossianum_DSM_10300**	**0.75**	**85.21**
**LAC_BACT02**	**OLB10**	**0.01**	**99.75**
**LAC_BACT03**	**Bacteria_BacteroidetesChlorobi_group_Bacteroidetes_Flavobacteriia_Flavobacteriales_Cryomorphaceae_Crocinitomix_catalasitica_ATCC_23190**	**0.68**	**86.51**
**LAC_PROT01**	**CG18_big_fil_WC_8_21_14_2_50_Hydrogenophilales_58_12**	**0.54**	**89.32**
**LAC_PROT02**	**LAC_PROT03**	**0.08**	**98.33**
**LAC_PROT03**	**LAC_PROT02**	**0.08**	**98.33**
**LAC_CLO01**	**Bacteria_CP_WWE1_candidate_division_WWE1_bacterium_JGI_0000039_M09_TAsludge_001_159**	**0.01**	**99.86**

LAC_CHLX02	UTCFX1	0.00	100.00
LAC_PROT04	Bacteria_Proteobacteria_Gammaproteobacteria_Alteromonadales_Alteromonadaceae_Melitea_salexigens_DSM_19753	0.43	91.44
LAC_GMT01	Bacteria_Gemmatimonadetes_Gemmatimonadetes_Gemmatimonadales_Gemmatimonadaceae_Gemmatimonas_aurantiaca_T_27	0.12	97.58
LAC_PROT05	LAC_PROT17	0.03	99.36
LAC_PROT06	LAC_PROT19	0.22	95.59
LAC_PLT01	Bacteria_Planctomycetes_Planctomycetia_Planctomycetales_Planctomycetaceae_Pirellula_staleyi_DSM_6068	0.61	88.05
LAC_PROT07	LAC_PROT20	0.08	98.41
LAC_PROT08	LAC_PROT20	0.27	94.68
LAC_ACD03	OLB17	0.18	96.39
LAC_IGN01	Bacteria_Ignavibacteria_RBG_16_Ignavibacteria_36_9	0.10	97.97
LAC_PROT09	UTPRO2	0.13	97.49
LAC_BACT04	UTBCD1	0.05	99.03
LAC_BACT05	Sphingobacteriia_bacterium_RIFOXYD2_FULL_35_12	0.35	93.06
LAC_D-T01	LAC_D-T02	0.01	99.87
LAC_SPR01	Bacteria_Spirochaetes_Spirochaetia_Spirochaetales_Leptospiraceae_Turneriella_parva_H_DSM_21527	0.25	95.08
LAC_ACT01	Bacteria_Actinobacteria_Actinobacteria_unclassified_Actinobacteria_Candidatus_Microthrix_parvicella_Bio17_1	0.41	91.84
LAC_BAC12	OLB21	1.22	75.94
LAC_BAC13	Bacteria_Armatimonadetes_Fimbriimonas_ginsengisoli_Gsoil_348	0.37	92.70
LAC_BAC14	Bacteria_Cyanobacteria_Oscillatoriales_Microcoleus_sp__PCC_7113	1.00	80.31
LAC_BACT06	CG18_big_fil_WC_8_21_14_2_50_Bacteroidetes_41_14	0.40	92.02
LAC_PROT10	LAC_PROT27	0.16	96.82
LAC_PROT11	Burkholderiales_bacterium_RIFCSPLOWO2_12_FULL_65_40	0.08	98.46
LAC_PROT12	LAC_PROT27	0.22	95.59
LAC_CHLX03	LAC_CHLX09	0.13	97.47
LAC_PROT13	Bacteria_Proteobacteria_Gammaproteobacteria_Thiothrichales_uncultured_SG8_50	0.28	94.54
LAC_PROT14	Bacteria_Proteobacteria_Betaproteobacteria_Hydrogenophilales_GWE1_Thiobacillus_62_9	0.10	98.12
LAC_IGN02	LAC_IGN05	0.03	99.39
LAC_IGN03	UTCHB2	0.16	96.84
LAC_IGN04	Bacteria_Ignavibacteria_GWA2_Ignavibacteriae_55_25	0.52	89.66
LAC_ARCH01	Archaea_Euryarchaeota_Methanomicrobia_Methanosarcinales_Methanosarcinaceae_Methanosarcina_barkeri_Fusaro_DSM_804	0.10	97.99
LAC_MIC01	Candidatus_Collierbacteria_bacterium_RIFOXYB1_FULL_49_13	0.75	85.11

**LAC_MIC02**	**LAC_MIC04**	**0.59**	**88.44**
**LAC_PROT15**	**Bacteria_Proteobacteria_deltaepsilon_subdivisions_Deltaproteobacteria_Myxococcales_Sorangiineae_Polyangiaceae_Chondromyces_apiculatus_DSM_436**	**0.35**	**93.10**
**LAC_PROT16**	**Bacteria_Proteobacteria_Alphaproteobacteria_Rhizobiales_Phyllobacteriaceae_Aquamicrobium_defluvii_W13Z1**	**0.08**	**98.32**
**LAC_MIC03**	**UTCPR1**	**0.00**	**100.00**
**LAC_BACT07**	**UTBCD1**	**0.05**	**98.99**
**LAC_IGN05**	**LAC_IGN02**	**0.03**	**99.39**
**LAC_VER01**	**LAC_VER02**	**0.09**	**98.18**
**LAC_VER02**	**LAC_VER01**	**0.09**	**98.18**
**LAC_PROT17**	**LAC_PROT05**	**0.03**	**99.36**
**LAC_PROT18**	**Bacteria_Proteobacteria_Gammaproteobacteria_Xanthomonadales_Xanthomonadaceae_Rudaea_cellulosilytica_DSM_22992**	**0.28**	**94.43**
**LAC_ACD04**	**Bacteria_Acidobacteria_RIFCSPLOWO2_02_FULL_Acidobacteria_68_18**	**0.40**	**92.19**
**LAC_BAC15**	**OLB6**	**0.00**	**99.96**
**LAC_BAC16**	**Bacteria_ChlamydiaeVerrucomicrobia_group_Verrucomicrobia_Spartobacteria_Chthoniobacter_flavus_Ellin428_unfinished_sequence**	**0.95**	**81.20**
**LAC_BAC18**	**Bacteria_Chloroflexi_RBG_16_Chloroflexi_68_14**	**0.74**	**85.42**
**LAC_BACT08**	**LAC_BACT13**	**0.66**	**87.00**
**LAC_IGN06**	**Bacteria_RIF-IGX_RIFOXYC2_FULL_RIF_IGX_35_21**	**0.59**	**88.31**
**LAC_BACT09**	**LAC_PROT25**	**0.00**	**100.00**
**LAC_CHLX04**	**LAC_CHLX08**	**0.11**	**97.80**
**LAC_CHLX05**	**CG2_30_FULL_Chloroflexi_64_16**	**0.32**	**93.76**
**LAC_PROT19**	**OLB2**	**0.00**	**99.99**
**LAC_OMN01**	**Bacteria_OP3X_RIFCSPHIGHO2_02_FULL_OP3X_63_14**	**0.49**	**90.34**
**LAC_VER03**	**Bacteria_ChlamydiaeVerrucomicrobia_group_Verrucomicrobia_Opitutae_Opitutales_Opitutaceae_Opitutus_terrae_PB90_1**	**0.18**	**96.48**
**LAC_VER04**	**Bacteria_ChlamydiaeVerrucomicrobia_group_Verrucomicrobia_Verrucomicrobiae_Verrucomicrobiales_Verrucomicrobia_subdivision_3_bacterium_Ellin514**	**0.46**	**90.90**
**LAC_PROT20**	**LAC_PROT07**	**0.08**	**98.41**
**LAC_BACT10**	**Bacteria_BacteroidetesChlorobi_group_Bacteroidetes_Sphingobacteriia_Sphingobacteriales_Chitinophagaceae_Niastella_koreensis_GR20_10_DSM_17620**	**0.31**	**93.92**
**LAC_BACT11**	**Bacteria_BacteroidetesChlorobi_group_Bacteroidetes_Sphingobacteriia_Sphingobacteriales_Saprospiraceae_Haliscomenobacter_hydrossis_O_DSM_1100**	**0.48**	**90.51**
**LAC_IGN07**	**UTCHB3**	**0.00**	**100.00**
**LAC_VER05**	**Bacteria_ChlamydiaeVerrucomicrobia_group_Verrucomicrobia_Spartobacteria_Chthoniobacter_flavus_Ellin428_unfinished_sequence**	**0.53**	**89.54**

LAC_PROT21	Bacteria_Proteobacteria_Alphaproteobacteria_unclassified_Alphaproteobacteria_Micavibrio_aeruginosavorus_ARL_13	0.65	87.09
LAC_PROT22	OLB13	0.00	99.99
LAC_BAC20	UTPRO1	0.00	100.00
LAC_BACT12	LAC_BACT02	0.40	92.12
LAC_NIT01	OLB3	0.00	99.94
LAC_CHLX06	CG2_30_FULL_Chloroflexi_64_16	0.63	87.65
LAC_PROT22	Gammaproteobacteria_bacterium_RIFCSPLOWO2_12_FULL_52_10	0.64	87.35
LAC_MIC04	LAC_MIC02	0.59	88.44
LAC_PROT23	LAC_PROT19	0.24	95.17
LAC_PLT02	UTAMX1	0.00	100.00
LAC_CHLX07	Bacteria_Chloroflexi_GWB2_Chloroflexi_54_36	0.40	92.09
LAC_BAC21	LAC_BAC20	0.61	88.00
LAC_BACT13	LAC_BACT08	0.66	87.00
LAC_PROT24	Betaproteobacteria_bacterium_RIFCSPLOWO2_12_FULL_62_13b	0.52	89.79
LAC_PROT25	LAC_BACT09	0.00	100.00
LAC_SCH01	CG_4_10_14_0_2_um_filter_Saccharibacteria_52_9	0.69	86.30
LAC_ACD05	LAC_ACD07	0.38	92.40
LAC_CHLX08	UTCFX2	0.00	99.99
LAC_ARM01	OLB18	0.00	99.95
LAC_MIC05	LAC_MIC04	0.81	83.95
LAC_PROT26	Bacteria_Proteobacteria_Alphaproteobacteria_Rhodobacterales_Rhodobacteraceae_Rhodovulum_sp__PH10	0.28	94.38
LAC_DADA01	Candidatus_Dadabacteria_bacterium_RIFCSPHIGHO2_12_FULL_53_21	0.25	95.11
LAC_CHLX09	LAC_CHLX03	0.13	97.47
LAC_CHLX10	UTCFX3	0.00	99.93
LAC_ACD06	Bacteria_Acidobacteria_RBG_13_Acidobacteria_68_16	0.33	93.48
LAC_ACT02	Actinobacteria_bacterium_RBG_16_68_12	0.31	93.96
LAC_ACT03	LAC_ACT01	0.58	88.52
LAC_ACT04	Bacteria_Actinobacteria_Actinobacteria_Actinobacteridae_Actinomycetales_Micrococcineae_Cellulomonadaceae_Actinotalea_ferrariae_CF5_4	0.17	96.58
LAC_BAC22	UTCHB1	0.00	100.00
LAC_BAC23	LAC_BAC08	0.84	83.47
LAC_BAC24	Bacteria_RIF-CHLX_GWC2_RIF_CHLX_73_18	0.40	92.09
LAC_PROT27	LAC_PROT10	0.16	96.82
LAC_CHLX11	Bacteria_Chloroflexi_Caldilineae_Caldilineales_Caldilineaceae_Caldilinea_a	0.22	95.58

	erophila_STL_6_O1_DSM_14535		
LAC_ACD07	LAC_ACD05	0.38	92.40
LAC_CHLX12	LAC_CHLX06	0.82	83.78
LAC_CHLX13	Bacteria_Chloroflexi_Anaerolineae_Anaerolineales_Anaerolineaceae_Anaerolinea_thermophila_UNI_1	0.28	94.43
LAC_CHLX14	OLB15	0.37	92.70
LAC_CHLX15	LAC_CHLX17	0.64	87.28
LAC_CHLX16	LAC_CHLX17	0.25	94.97
LAC_CHLX17	LAC_CHLX16	0.25	94.97
LAC_PROT28	LAC_PROT08	0.59	88.31
LAC_CHLX18	Bacteria_RIF-CHLX_RBG_16_RIF_CHLX_72_14	0.14	97.17
LAC_PROT29	Rhodobacteraceae_bacterium_GWF1_65_7	0.28	94.38
LAC_PROT30	UTPRO2	0.00	99.97
LAC_MIC06	Candidatus_Roizmanbacteria_bacterium_RIFOXYA1_FULL_41_12	0.70	86.25
LAC_D-T02	LAC_D-T01	0.01	99.87
OLB1	Candidatus_Brocadia_sinica_JPN1	0.00	99.90
OLB10	LAC_BACT02	0.01	99.75
OLB11	Bacteria_BacteroidetesChlorobi_group_Bacteroidetes_Sphingobacteriia_Sphingobacteriales_Chitinophagaceae_Chitinophaga_pinensis_DSM_2588	0.41	91.93
OLB12	Bacteria_BacteroidetesChlorobi_group_Bacteroidetes_Cytophagia_Cytophagales_Flammeovirgaceae_Fulvivirga_imtechensis_AK7	0.39	92.33
OLB13	LAC_PROT22	0.00	99.99
OLB14	Bacteria_Chloroflexi_RBG_16_Chloroflexi_51_16	0.32	93.67
OLB15	LAC_CHLX14	0.37	92.70
OLB16	LAC_BAC05	0.00	99.92
OLB17	LAC_ACD03	0.18	96.39
OLB18	LAC_ARM01	0.00	99.95
OLB2	LAC_PROT19	0.00	99.99
OLB20	OLB21	0.41	91.82
OLB21	OLB20	0.41	91.82
OLB22	Candidatus_Roizmanbacteria_bacterium_RIFCSPHIGHO2_01_FULL_39_12b	1.04	79.49
OLB23	Candidatus_Roizmanbacteria_bacterium_RIFCSPLOWO2_01_FULL_38_11	0.62	87.77
OLB3	LAC_NIT01	0.00	99.94
OLB4	LAC_BAC22	0.00	99.99
OLB5	UTCHB1	0.62	87.78

**OLB6**	**LAC_BAC15**	**0.00**	**99.96**
**OLB7**	**Bacteria_RIF-IGX_RIFOXYC2_FULL_RIF_IGX_35_21**	**0.87**	**82.83**
**OLB8**	**OLB9**	**0.58**	**88.57**
**OLB9**	**OLB8**	**0.58**	**88.57**
**UTAMX1**	**LAC_PLT02**	**0.00**	**100.00**
**UTAMX2**	**UTAMX1**	**0.16**	**96.76**
**UTBCD1**	**LAC_BACT04**	**0.05**	**99.03**
**UTCFX1**	**LAC_CHLX02**	**0.00**	**100.00**
**UTCFX2**	**LAC_CHLX08**	**0.00**	**99.99**
**UTCFX3**	**LAC_CHLX10**	**0.00**	**99.93**
**UTCFX4**	**LAC_CHLX01**	**0.12**	**97.66**
**UTCFX5**	**LAC_PROT22**	**0.00**	**99.99**
**UTCHB1**	**LAC_BAC22**	**0.00**	**100.00**
**UTCHB2**	**LAC_IGN05**	**0.05**	**99.10**
**UTCHB3**	**LAC_IGN07**	**0.00**	**100.00**
**UTCPR1**	**LAC_MIC03**	**0.00**	**100.00**
**UTPLA1**	**Bacteria_Plantomycetes_Phycisphaerae_uncultured_SMTZ_30**	**0.95**	**81.25**
**UTPRO1**	**LAC_BAC20**	**0.00**	**100.00**
**UTPRO2**	**LAC_PROT30**	**0.00**	**99.97**

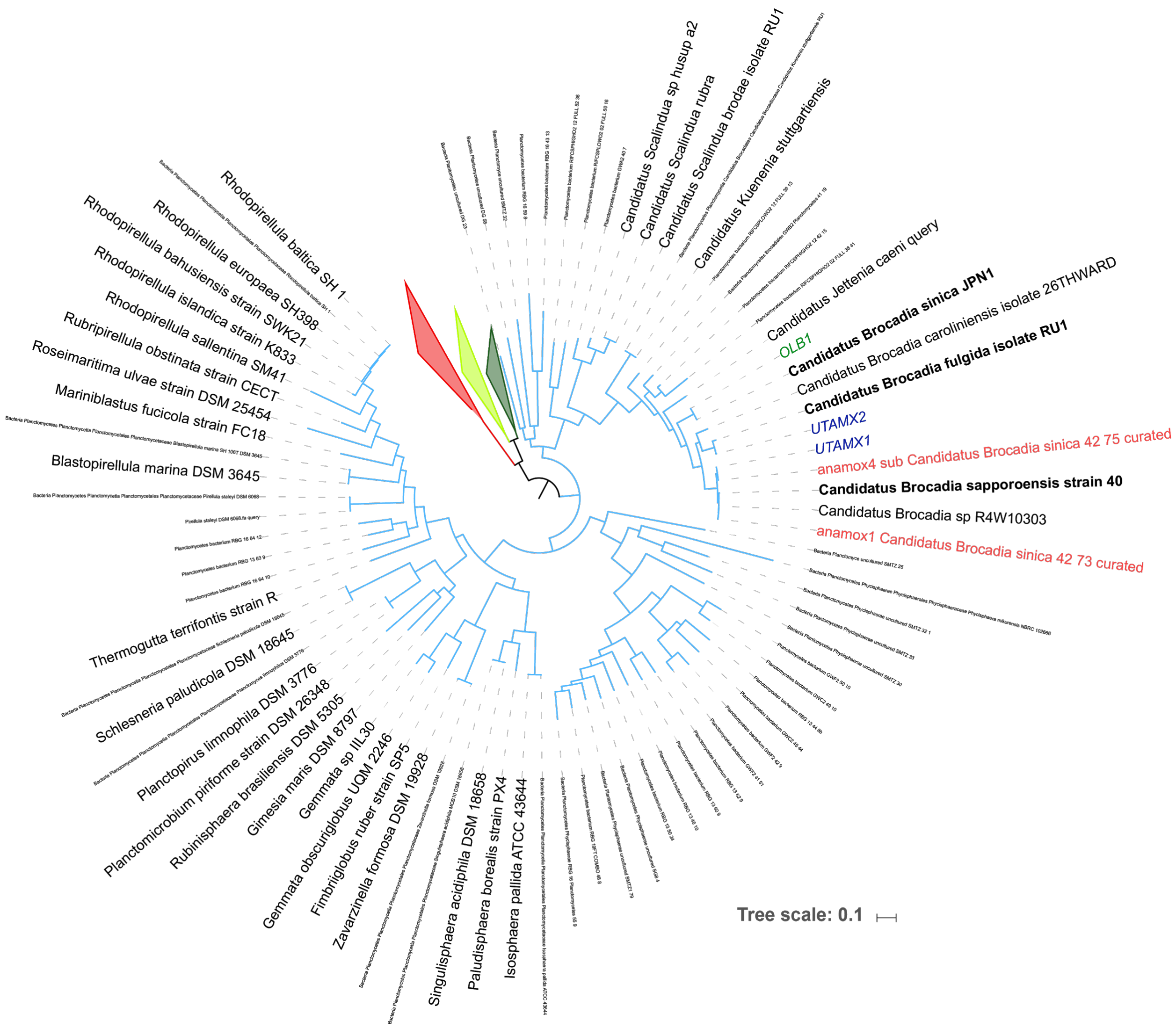

Figure S3 Maximum likelihood tree of the PVC clade (*Planctomycetes*, *Verrucomicrobia*, *Chlamydiae*, and *Lentisphaerae*). This tree is based on amino acid sequence alignment of 15 concatenated Ribosomal Proteins. Genomes from the current study, genomes from previous studies, and all reference sequences were used for the construction of the tree. To these sequences, 35 *Planctomycetes* were added from NCBI, bringing the total number of sequences to 123. Sequences were aligned with MAFFT (default parameters) and a RaxML tree was constructed in The CIPRES Science Gateway V. 3.3. Branch colors follow the ggkbase phyla color palette. The *Chlamydiae*, *Verrucomicrobia*, and *Lentisphaerae* phyla are collapsed to allow easier viewing of the *Planctomycetes*. Genomes of anammox bacteria from the current paper and previous papers are colored according to marking in Figure 2A. *Planctomycetes* genomes from NCBI are in larger font, and closest NCBI references to the anammox bioreactor's genomes are in bold. All anammox bacteria are of the genus *Brocadia*. The anammox bacterium from the current study (both strains are presented) and the dominant anammox bacterium from Lawson et al. are both closely related to *Brocadia sapporoensis*. The second anammox bacterium from the Lawson et al. study is closely related to *Brocadia fulgida*. The anammox bacterium from the Speth et al. study is closely related to *Brocadia sinica*
.

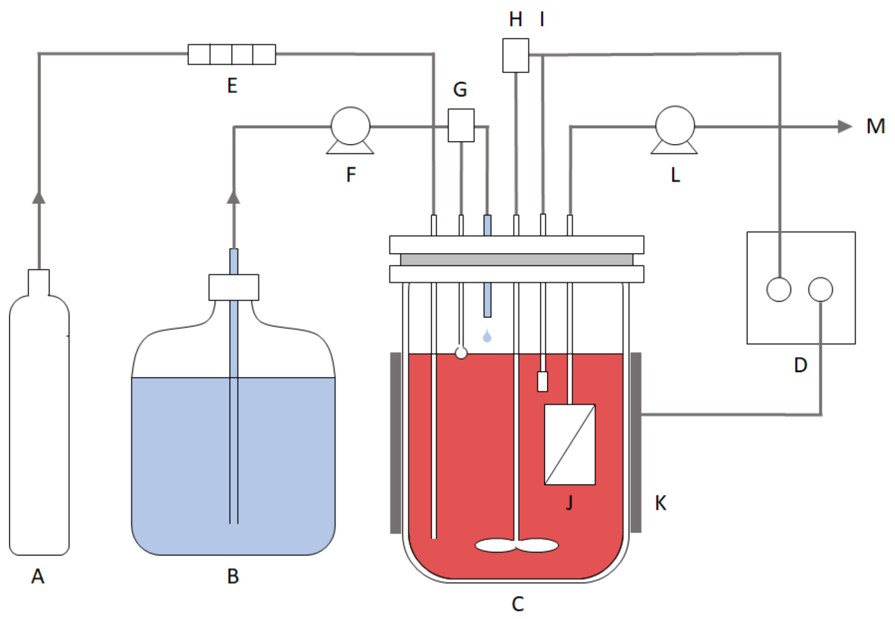

Figure S4 Schematic of the anaerobic membrane bioreactor. The components of the anaerobic membrane bioreactor include: A, influent gas tank; B, influent media tank; C, 1L reactor vessel; D, power supply device; E, flowmeter; F, influent peristaltic pump; G, level controller; H, impeller; I, temperature probe; J, membrane module; K, heating jacket; L, effluent peristaltic pump; and M, effluent line.

Table S8 Composition of the synthetic wastewater feed. This recipe, based on van de Graaf et al. 1995, was modified to better mimic the composition of the anaerobic digester sidestream at East Bay Municipal Utility District (Oakland, CA). Concentrations of nitrogen species varied with time, (Figure 3). Additionally, the concentrations of the four constituents asterisked below--iron, zinc, molybdenum, and copper--were increased from their lower to higher values on Day 353.

Constituent	Concentration	Unit
$(NH_4)_2SO_4$	40-500	mg-N/L
$NaNO_2$	5-660	mg-N/L
NaCl	1000	mg/L
$MgCl_2 \cdot 6H_2O$	500	mg/L
KH_2PO_4	200	mg/L
KCl	300	mg/L
$CaCl_2 \cdot 2H_2O$	180	mg/L
$KHCO_3$	420	mg/L
*$FeCl_2 \cdot 4H_2O$	7.7; 17.9	mg/L
$CoCl_2 \cdot 6H_2O$	0.24	mg/L
$MnCl_2 \cdot 4H_2O$	0.99	mg/L
*$ZnCl_2$	0.07; 0.20	mg/L
H_3BO_3	0.014	mg/L
*$Na_2MoO_4 \cdot 2H_2O$	0.10; 0.22	mg/L
$NiCl_2 \cdot 6H_2O$	0.19	mg/L
*$CuCl_2 \cdot 2H_2O$	0.002; 0.17	mg/L
$Na_2SeO_3 \cdot 5H_2O$	0.16	mg/L
pH	6.8-7.0	-

Supplemental methods

Refinement of the *Candidatus Brocadia* sp. genome.

After running dRep but before running ra2, we discovered that the hydrazine synthase (Hzs) gene, fundamental to the anammox process, was not binned with the *Brocadia* sp. genome. The Hzs gene was skewed in both GC and CV, but the taxonomic identification of the scaffold linked it to the *Brocadia* sp. genome. No other Planctomycetes genomes were found in the GC and CV ranges that covered the Hzs-harboring scaffold.

To test if the Hzs gene belonged to the *Brocadia* sp. genome, we clustered scaffolds according to their tetramer frequency based on Emergent Self-Organizing Maps (ESOMs) with Databionic ESOM Analyzer [59]. In summary, we downloaded the *Brocadia* sp. genome, a temporary bin with the Hzs gene, and a second temporary bin with all other *Planctomycetes* scaffolds within the GC and CV ranges of both prior bins. To each of these bins we added representative genomes with varying CV values, and also 12 *Brocadiales* genomes found in NCBI Genome database. The *Brocadia* sp. genome, along with the two temporary bins, clustered with *Brocadia sinica*. Following this analysis, both the Hzs bin and the tmp bin were added to the *Brocadia* sp. bin.

Table S9 Replication rates of genomes for each time point, calculated using the iRep program.

Genome	D0	D82	D166	D284	D328	D437
LAC_BAC01	1.46	NA	1.44	NA	NA	NA
LAC_BAC02	1.40	NA	NA	NA	NA	NA
LAC_BAC03	1.30	NA	1.42	NA	NA	NA
LAC_BAC04	1.21	NA	1.30	NA	NA	NA
LAC_BAC05	1.34	NA	1.40	NA	NA	NA
LAC_CHLX01	1.31	1.40	1.16	1.32	1.11	1.21
LAC_BAC06	1.25	NA	1.28	NA	NA	NA
LAC_BAC08	1.31	NA	1.38	NA	NA	NA
LAC_BAC09	1.36	NA	1.40	NA	NA	NA
LAC_BAC11	1.28	NA	1.30	NA	NA	NA
LAC_BACT02	1.32	1.44	1.39	NA	NA	NA
LAC_BACT03	1.30	1.21	1.33	NA	1.37	NA
LAC_PROT02	1.15	1.18	1.17	1.30	1.30	NA
LAC_PROT03	1.27	1.31	1.32	1.40	1.36	NA
LAC_CHLX02	1.31	1.31	1.16	1.17	1.43	NA
LAC_PROT04	1.42	NA	NA	NA	NA	NA
LAC_GMT01	1.34	NA	1.39	NA	NA	NA
LAC_PROT05	1.67	NA	NA	NA	NA	NA
LAC_PROT06	1.24	NA	1.29	NA	NA	NA
LAC_PROT07	1.55	NA	1.62	NA	NA	NA
LAC_PROT08	1.45	NA	1.53	NA	NA	NA
LAC_ACD03	1.36	1.13	1.19	1.35	1.29	NA
LAC_PROT09	1.41	NA	NA	NA	NA	NA
LAC_BACT04	1.31	1.32	1.26	1.26	1.18	1.28
LAC_BACT05	1.41	NA	NA	NA	NA	NA
LAC_SPR01	1.37	NA	NA	NA	NA	NA
LAC_BAC13	NA	1.35	NA	NA	NA	NA
LAC_BACT06	NA	1.37	NA	NA	NA	NA
LAC_PROT11	NA	1.35	NA	NA	NA	NA
LAC_PROT12	NA	1.29	NA	NA	NA	NA
LAC_CHLX03	NA	1.36	NA	NA	NA	NA
LAC_PROT13	NA	1.28	NA	NA	NA	NA
LAC_PROT14	NA	1.32	NA	NA	NA	NA
LAC_IGN02	NA	1.27	1.81	NA	NA	NA
LAC_IGN03	NA	1.46	NA	NA	NA	NA
LAC_IGN04	NA	1.29	NA	NA	NA	NA
LAC_ARCH01	NA	1.31	NA	NA	NA	NA

LAC_MIC01	NA	1.47	NA	NA	1.42	1.36
LAC_MIC02	NA	1.39	NA	NA	2.57	2.40
LAC_PROT16	NA	1.17	NA	NA	NA	NA
LAC_MIC03	NA	1.54	1.60	NA	NA	NA
LAC_BACT07	NA	1.31	NA	NA	NA	NA
LAC_IGN05	1.42	1.31	1.38	1.51	1.58	1.90
LAC_VER01	NA	1.38	NA	NA	NA	NA
LAC_VER02	1.34	1.37	1.38	NA	1.44	NA
LAC_PROT17	NA	1.28	NA	NA	NA	NA
LAC_PROT18	NA	1.51	NA	NA	1.56	NA
LAC_BAC16	NA	NA	1.36	NA	NA	NA
LAC_BAC17	NA	NA	1.25	NA	NA	NA
LAC_BAC18	1.37	NA	1.34	NA	NA	NA
LAC_BACT08	1.22	NA	1.30	NA	NA	NA
LAC_IGN06	NA	1.41	1.33	NA	1.36	NA
LAC_PROT19	1.17	1.38	1.22	1.32	1.38	NA
LAC_OMN01	NA	NA	1.25	NA	NA	NA
LAC_PROT20	1.27	NA	1.31	NA	1.38	NA
LAC_BACT10	1.76	NA	NA	NA	NA	NA
LAC_BACT11	1.37	NA	1.32	1.32	NA	NA
LAC_IGN07	1.29	NA	1.30	1.48	1.39	1.41
LAC_VER05	NA	NA	1.33	NA	NA	NA
LAC_PROT21	NA	NA	NA	1.29	1.27	1.16
LAC_PROT22	NA	NA	NA	1.38	1.24	1.40
LAC_BAC20	1.13	1.35	1.13	1.19	1.14	1.21
LAC_BACT12	1.16	NA	1.14	1.18	1.19	NA
LAC_NIT01	NA	NA	1.25	1.31	1.41	NA
LAC_CHLX06	1.24	NA	1.21	1.28	1.18	1.28
LAC_PROT22	1.41	NA	1.36	1.24	1.23	NA
LAC_MIC04	NA	NA	NA	1.35	1.23	NA
LAC_PROT23	NA	NA	NA	1.47	NA	NA
LAC_PLT02	NA	1.56	2.13	1.07	1.13	1.12
LAC_CHLX07	NA	NA	1.69	NA	1.35	1.71
LAC_BAC21	1.12	1.21	1.13	1.25	1.23	NA
LAC_BACT13	NA	NA	NA	NA	1.31	NA
LAC_PROT25	NA	NA	NA	1.52	1.43	NA
LAC_ACD05	1.55	NA	NA	NA	1.48	1.50
LAC_CHLX08	NA	NA	NA	NA	1.38	NA
LAC_ARM01	NA	NA	NA	NA	1.28	1.31
LAC_MIC05	NA	NA	NA	NA	1.22	NA

LAC_PROT26	NA	NA	NA	NA	1.29	1.26
LAC_CHLX09	1.74	1.45	1.56	1.63	1.42	1.46
LAC_CHLX10	1.72	1.37	1.26	1.42	1.36	1.59
LAC_ACD06	NA	NA	NA	NA	NA	1.27
LAC_ACT02	NA	NA	NA	NA	1.30	1.29
LAC_ACT03	NA	NA	NA	NA	1.29	1.36
LAC_ACT04	NA	NA	NA	NA	1.39	1.26
LAC_BAC22	NA	1.34	NA	1.39	1.20	1.26
LAC_BAC23	1.27	1.14	1.17	1.17	1.18	1.19
LAC_BAC24	NA	NA	NA	NA	1.37	1.39
LAC_PROT27	2.05	2.11	2.06	1.56	1.34	1.26
LAC_CHLX11	NA	NA	NA	NA	NA	1.29
LAC_CHLX12	NA	NA	NA	NA	NA	1.23
LAC_CHLX13	NA	NA	NA	NA	NA	1.48
LAC_CHLX14	NA	NA	NA	NA	NA	1.33
LAC_CHLX15	NA	NA	NA	NA	NA	1.31
LAC_CHLX16	NA	NA	NA	NA	1.30	1.29
LAC_CHLX17	NA	NA	NA	NA	1.35	1.25
LAC_PROT28	NA	NA	1.33	1.27	1.20	1.19
LAC_CHLX18	NA	NA	NA	NA	1.41	1.36
LAC_PROT30	NA	NA	NA	1.89	1.67	1.43
LAC_MIC06	NA	NA	NA	NA	NA	1.15
LAC_D-T02	NA	1.59	NA	1.59	1.43	1.42
